# Advanced Glycation End-Products (AGEs) and Their Soluble Receptor (sRAGE) in Women Suffering from Systemic Lupus Erythematosus (SLE)

**DOI:** 10.3390/cells10123523

**Published:** 2021-12-13

**Authors:** Agnieszka Nowak, Brygida Przywara-Chowaniec, Aleksandra Damasiewicz-Bodzek, Dominika Blachut, Ewa Nowalany-Kozielska, Krystyna Tyrpień-Golder

**Affiliations:** 1 Department of Chemistry, Faculty of Medical Sciences in Zabrze, Medical University of Silesia, 40-055 Katowice, Poland; aleksandra.bodzek@sum.edu.pl (A.D.-B.); ktyrpien@sum.edu.pl (K.T.-G.); 2 2nd Department of Cardiology, Faculty of Medical Sciences in Zabrze, Medical University of Silesia, 40-055 Katowice, Poland; bprzywara-chowaniec@365.sum.edu.pl (B.P.-C.); d201036@365.sum.edu.pl (D.B.); ekozielska@365.sum.edu.pl (E.N.-K.)

**Keywords:** systemic lupus erythematosus (SLE), advanced glycation end products (AGEs), carboxymethyllysine (CML), carboxyethyllysine (CEL), pentosidine, soluble receptor for advanced glycation end-products (sRAGE)

## Abstract

Systemic lupus erythematosus (SLE) is characterized by abnormal action of the immune system and a state of chronic inflammation. The disease can cause life-threatening complications. Neoepitopes arising from interdependent glycation and oxidation processes might be an element of SLE pathology. The groups included in the study were 31 female SLE patients and 26 healthy female volunteers (the control group). Blood serum samples were obtained to evaluate concentrations of advanced glycation end-products (AGEs), carboxymethyllysine (CML), carboxyethyllysine (CEL), pentosidine, and a soluble form of the receptor for advanced glycation end-products (sRAGE). Compared to a healthy control group, the SLE patients exhibited a higher concentration of AGEs and a lower concentration of sRAGE in serum. There were no statistically significant differences in serum CML, CEL, and pentosidine concentrations between the groups. Therefore, SLE patients could be at risk of intensified glycation process and activation of the proinflammatory receptor for advanced glycation end-products (RAGE), which could potentially worsen the disease course; however, it is not clear which compounds contribute to the increased concentration of AGEs in the blood. Additionally, information about the cigarette smoking and alcohol consumption of the study participants was obtained.

## 1. Introduction

Systemic lupus erythematosus (SLE) is a widely studied disease; however, its direct causes are unknown. The occurrence and prevalence of SLE vary among different populations and are much higher for women than for men [1,2]. The role of female reproductive hormones in the development of the disease is being studied [3]. Polymorphisms and mutations in numerous gene loci are linked to the etiopathogenesis of SLE, e.g., the genes of the major histocompatibility complex, complement system, nucleic acid metabolism, and the receptor for advanced glycation end-products (RAGE) [4,5]. Airborne pollutants and cigarette smoke are examples of environmental factors suspected to cause SLE development [6,7]. The simultaneous presence of genetic, epigenetic, and environmental factors most likely leads to the development of SLE [8].

The characteristic feature of SLE is the presence of autoantibodies, especially antinuclear antibodies such as anti-dsDNA [9,10]. Therefore, the disease is characterized by the abnormal action of the immune system and a state of chronic inflammation. Immune complexes are deposited in various tissues [9,11]. SLE contributes to life-threatening complications, such as renal failure, seizures, and accelerated arteriosclerosis [12,13].

Chronic inflammation in SLE appears to be linked to an intensified glyco-oxidation process (also known as the glycation process) [11,14]. Advanced glycation end-products (AGEs) are formed during this complex reaction and can be recognized by the immune system as neoepitopes [15,16,17]. The initial phase of glyco-oxidation begins with the reaction of a carbonyl compound (e.g., glucose or other monosaccharides, glyoxal, methylglyoxal) and an amino group (of either free amino acids or a protein). Subsequently, numerous reactions may occur, such as rearrangement, cross-linking, cyclization, isomerization, and cleavage [18,19]. Oxidative conditions and carbonyl stress promote AGEs formation [20]. Various AGEs are found in body fluids, cells, and tissues of human subjects, including carboxymethyllysine (CML), carboxyethyllysine (CEL), pentosidine, and many more [18]. Studies show a prominent relationship between enhanced AGEs formation and various diseases, including systemic lupus erythematosus (SLE) [20,21,22,23,24,25,26]. Current findings on the concentrations of AGEs in SLE patients are sparse. To date, the total concentration of AGEs in blood samples has been evaluated in two studies (using ELISA assay) [14,22]. Two more studies used the skin autofluorescence method [11,27]. Only one study included a group consisting solely of women (*n* = 9) [11]. Regardless of the method used, increased concentrations of AGEs among SLE patients compared to the healthy control group were noted in all these studies [11,14,22,27].

Unfortunately, it is not known which specific compounds contribute to the overall increase of AGEs concentration in SLE patients. Despite the wide range of compounds participating in the glycation process, there are few available studies focused on the evaluation of concentrations of individual compounds in SLE patients. These include studies concerning CML (*n* = 9), CEL (*n* = 9 and *n* = 40), pentosidine (*n* = 37 and *n* = 82), fructosamine (*n* = 37), and an unidentified product of ribose and protein reaction (*n* = 40) [11,14,21,22,27,28]. A marginal increase in fructosamine concentration and contradictory results related to pentosidine concentration in the blood serum of SLE patients have been noted so far [14,28].

Advanced glycation end-products can affect the structure of proteins (through covalent peptide cross-linking) [29] and activate RAGE [30,31,32]. These phenomena cause impairment of cells, extracellular matrix, and tissue functions [29,30,31,32]. It is suggested that RAGE plays a role in nucleic acid recognition by the immune system [16]. Activated RAGE induces a proinflammatory response [15]. A soluble form of RAGE (sRAGE) binds to the ligands of RAGE, preventing activation of RAGE and therefore counteracting inflammation and cell death [33].

Therefore, this study aimed to evaluate concentrations of total AGEs, CML, CEL, pentosidine, and sRAGE simultaneously in serum samples of women suffering from SLE and compare them to a healthy control group. This approach increased the homogeneousness of the examined groups and enabled analysis of correlations between these parameters and clinical parameters.

## 2. Materials and Methods

### 2.1. Human Subjects

The subjects of the study were recruited between October 2017 and October 2018 during routine cardiological checks at the 2nd Department of Cardiology, Faculty of Medical Sciences in Zabrze, Medical University of Silesia. The SLE patients group consisted of 31 adult female patients who fulfilled the 1997 American College of Rheumatology (ACR) SLE classification criteria. Patients with diabetes and/or abnormal concentrations of glucose in their blood were excluded from the study. A total of 26 age-matched, healthy female volunteers (the control group) were included in the study. 

Blood samples were collected from patients on fasting and were allowed to clot at room temperature. Blood serum was obtained by centrifugation. Samples were stored at −80 °C until used. In addition, an original questionnaire was used to obtain information about exposure to cigarette smoke, smoking habits, alcohol use habits, and medication used against SLE.

The Local Bioethics Committee of the Medical University of Silesia agreement was obtained before the study.

### 2.2. ELISA Assay

The enzyme-linked immunosorbent assay (ELISA) method was used to evaluate concentrations of AGEs, CML, CEL, pentosidine, and sRAGE in the samples. The following ELISA kits were used during the study:

- OxiSelect™ Advanced Glycation End Product (AGE) Competitive ELISA Kit, catalogue number STA-817 (Cell Biolabs, Inc., San Diego, CA, USA) sensitivity 0.39 μg/mL; precision measured as coefficient of variation < 5% (intra-assay), <10% (inter-assay);
- OxiSelect™ N-epsilon-(Carboxymethyl) Lysine (CML) Competitive ELISA Kit, catalogue number STA-816 (Cell Biolabs, Inc., San Diego, CA, USA) sensitivity 0.05 μg/mL; precision measured as coefficient of variation < 6% (intra-assay), <10% (inter-assay);
- OxiSelect™ N-epsilon-(Carboxyethyl) Lysine (CEL) Competitive ELISA, catalogue number STA-813 (Cell Biolabs, Inc., San Diego, CA, USA) sensitivity 0.1 μg/mL; precision measured as coefficient of variation < 6% (intra-assay), <10% (inter-assay);
- PTD (Pentosidine) ELISA Kit, catalogue number E-EL-0091 (Elabscience, Houston, TX, USA) sensitivity 0.47 ng/mL; precision measured as coefficient of variation < 6% (intra-assay and inter-assay);
- RayBio^®^ Human RAGE ELISA Kit, catalogue number ELH-RAGE (RayBiotech, Norcross, GA, USA) sensitivity 3 pg/mL; precision measured as coefficient of variation < 10% (intra-assay), <12% (inter-assay).

The protocols delivered by the manufacturers were carefully followed to obtain the results. The measurements of absorbances were taken with Power Wave XS (BioTek, Winooski, VT, USA) microplate spectrophotometer, wavelength set to 450 nm. The data were processed using KCJunior, version 1.41.3 (BioTek, Winooski, VT, USA) software. 

Statistical analysis was performed with STATISTICA for Windows, version 13 (StatSoft) software. The distribution of data was measured using the Shapiro–Wilk test. Independent data between the group of SLE patients and the control group were compared using non-parametric Kolmogorov–Smirnov and Mann–Whitney U tests. To explore the statistical dependence between two variables, Spearman’s rank correlation coefficients were calculated. *p* < 0.05 was considered statistically significant.

## 3. Results

### 3.1. Characteristics of the Human Subjects

The basic characteristics of the groups included in the study are shown in Table 1. All the SLE patients and control group members were women of Caucasian descent.

### 3.2. ELISA Assay Results

**Figure 1:**
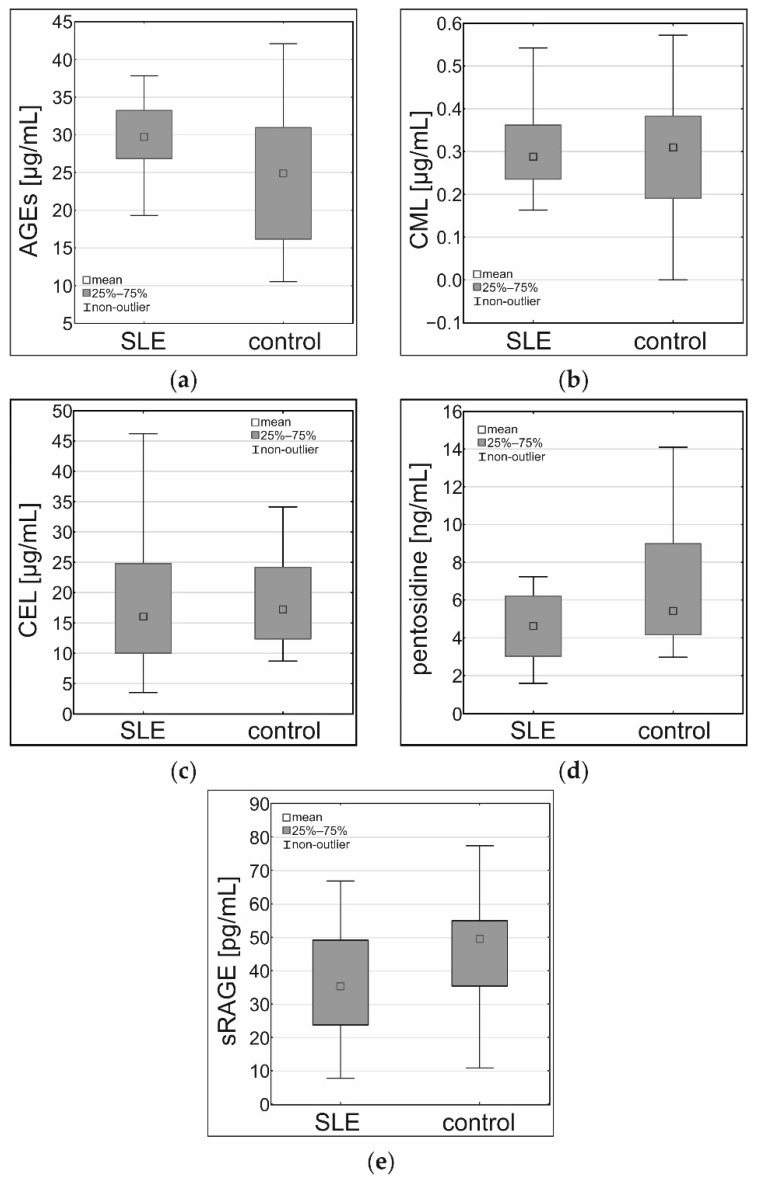
Results of the ELISA assay in the serum samples of the SLE patients and the control group: (**a**) concentrations of AGEs (µg/mL), (**b**) concentrations of CML (µg/mL), (**c**) concentrations of CEL (µg/mL), (**d**) concentrations of pentosidine (ng/mL), (**e**) concentrations of sRAGE (pg/mL).

There were statistically significant differences between AGEs and sRAGE concentrations in the SLE patients and the control group. AGEs concentration was higher in the SLE patients group (*p* < 0.01), as shown in Table 2 and Figure 1. The sRAGE concentration was lower in the SLE patients group (*p* < 0.05), as shown in Table 2 and Figure 1. Serum CML, CEL, and pentosidine did not exhibit a statistically significant difference when comparing the SLE patients to the control group (Table 2, Figure 1). However, the difference in pentosidine concentration in serum showed a trend toward significance and a tendency to be higher in the control group.

### 3.3. Correlation of the Data

There were no statistically significant correlations between the examined parameters in the control group. However, in the SLE patients group, there was a moderate positive correlation between the CEL and the pentosidine serum concentrations (R = 0.53, *p* < 0.01). The AGEs concentration correlated weakly and positively with smoking duration measured in years (R = 0.35; *p* < 0.05) but did not correlate with the age of all study participants analysed collectively (R = 0.25; *p* = 0.11). None of the examined parameters correlated with creatinine concentration, GFR, or SLEDAI-2K score (*p* > 0.05).

## 4. Discussion

As shown in Table 2, the concentration of serum AGEs is higher in the SLE patients when compared to the control group. These results agree with the available references [11,14,22,27].

**Figure 2:**
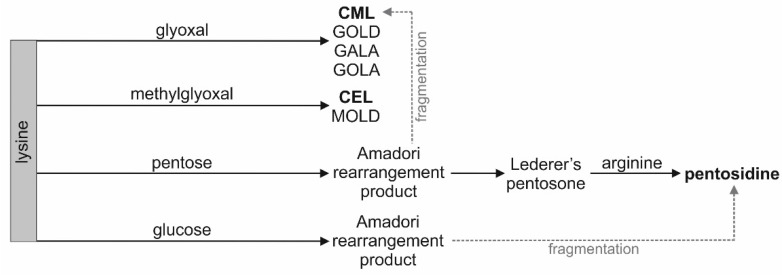
Simplified scheme of pathways leading to the formation of CML, CEL, and pentosidine (bold text). GALA—glycolic acid lysine amide, GOLA—glyoxal lysine amide, GOLD—glyoxal lysine dimer, MOLD—methylglyoxal lysine dimer.

As shown in Figure 2, CML and CEL are formed in the reaction of lysine with glyoxal and methylglyoxal, respectively. There are multiple sources of glyoxal and methylglyoxal in vivo: lipid peroxidation, oxidation of carbohydrates and ascorbic acid, degradation of glycated proteins, and metabolism of amino acids and ketone bodies [18,19,34]. There are two known pathways of pentosidine formation in vivo. The compound is formed during fragmentation of Amadori products arising from the reaction of glucose and lysine or during rearrangement of Lederer’s pentosone arising from pentose and lysine in oxidative conditions [18]. Lipid peroxidation and oxidative stress are intensified in SLE patients [35,36,37].

Therefore, it could be hypothesized that concentrations of particular AGEs are increased in the blood samples of the SLE patients. In fact, there were no statistically significant differences in concentrations of serum CML, CEL, and pentosidine between the groups. What is more, pentosidine concentration exhibited a near-significant tendency to be lower in the serum samples of the SLE patients. In two previous studies, no difference in pentosidine concentrations between the SLE patients and the healthy control was noted [21], and a higher concentration of pentosidine in the SLE patients was noted [14].

Considering the increase in total AGEs concentration in the serum samples, it is unclear which compounds contribute to this phenomenon. CML and CEL concentrations did not reflect the total concentration of AGEs in blood, as suggested by some authors [11]. These compounds cannot be used as markers of the glycation process in SLE patients, despite belonging to AGEs. Pathways leading to other AGEs, such as glycolic acid lysine amide (GALA), glyoxal lysine amide (GOLA), glyoxal lysine dimer (GOLD), and methylglyoxal lysine dimer (MOLD), should be investigated [18].

The concentration of CEL in the serum of the SLE patients correlates moderately and positively with the concentration of pentosidine. However, no common biosynthesis pathway for these two compounds is known (Figure 2). The theoretical possibility of the formation of pentosidine with methylglyoxal as substrate was described. Methylglyoxal is also a substrate of CEL [38]. It was noted that incubation of myoglobin with methylglyoxal resulted in the increased formation of pentosidine [39]. 

In this study, a decrease in sRAGE concentration in the serum samples of women suffering from SLE compared to the control group was observed. These results agree with most of the available data on the subject [11,14,22,40,41,42,43,44,45,46]. There are two possible explanations for the simultaneous decrease in the sRAGE concentration and increase in AGEs concentration. The deficit of sRAGE could be a primary phenomenon, allowing more AGEs to stay unbound in body fluids. It is also possible that the deficit is a secondary phenomenon, as the amount of sRAGE could be depleted by excessively generated AGEs or other ligands of this receptor. Regardless of the cause, the deficit of sRAGE might contribute to more frequent interactions between AGEs and transmembrane RAGE. It was observed that sRAGE administered in the murine model may prevent the activation of proinflammatory pathways [47]. Importantly, RAGE is linked to the process of nucleic acid internalization and immune response to nucleic acids. RAGE-deficient mice showed reduced inflammatory response to DNA in lungs [16]. SLE is characterized by the presence of autoantibodies, including antibodies against dsDNA [9,10].

In this study, 80.65% of the SLE patients suffered from cardiovascular disorders. Hypertension was the most prevalent (56.25%; *n* = 18), followed by coronary artery disease (15.63%; *n* = 5), valvular heart disease (12.50%; *n* = 4), and arteriosclerosis (9.38%; *n* = 3). There were single cases of aneurysm, vertebrobasilar insufficiency, hypotension, heart failure, venous insufficiency, and arrhythmia present in the group. Interestingly, the prevalence of hypertension varies in different studies, ranging from 9.4% to 77% in SLE patients. The lowest prevalence reported in a group consisting of 100% female SLE patients is 29.7%. These data refer to patients younger than the patients included in our study [48]. Cardiovascular disorders are a common implication of SLE, and the disease greatly increases the risk of cardiovascular events. Arteriosclerosis is accelerated in SLE and present in 28–40% of SLE patients, while abnormal perfusion in myocardium is present in 38% of the patients [48,49]. It should be noted that the major limitation of the study was the size of the examined groups, especially in the case of the evaluation of smoking habits. The high prevalence of cardiovascular disease could have affected the results too.

Among the women suffering from SLE, more patients declared smoking cigarettes regularly (at least one cigarette per day) in the past or present (Table 1), when compared to the healthy control. Interestingly, only in the SLE patient group were there persons (*n* = 2) who reported smoking more than 10 cigarettes per day (Figure S1). Considering the known role of smoking in the etiopathogenesis of SLE, this is a disadvantageous situation. What is more, tobacco smoke increases skin autofluorescence, which is a marker of AGEs accumulation in skin [50]. There was no difference between the groups in the number of persons declaring passive smoking, nor in smoking duration. However, it should be noted that there were few smokers in the healthy control group, which limits the significance of the statistical analysis. In the study, cumulative analysis of all participants in both groups showed a weak, positive correlation between the concentration of AGEs in serum and duration of smoking (but no correlation between AGEs concentration and age of the participants). Therefore, smoking might contribute to the increased accumulation of AGEs. Tobacco smoke has an undoubtedly negative impact on the organism and is linked to the formation of reactive oxygen species that can modify the structure of nucleic acids [51]. Additionally, concentrations of DNA oxidative damage markers are increased in patients with SLE [52]. Tobacco smoke enhances the expression of RAGE in pulmonary epithelia and causes an increase in amount of its ligands in mice. Simultaneous exposition to smoke and AGEs disrupts intracellular signalling pathways [53].

Fewer members of the SLE patients group reported drinking alcohol than of the healthy control. However, this difference is situated at the threshold of statistical significance (Table 1). Some studies suggest that moderate alcohol consumption might be protective against SLE, while others do not agree with this statement [54,55,56,57]. Considering the intake of medication against SLE by the majority of the patients and inconclusive data about the link between alcoholic beverages and the risk of SLE, alcohol consumption is not recommended.

There is a clear relationship between glycation and oxidation processes [18,20]. The chronic inflammation present in SLE enhances both processes, resulting in the formation of various AGEs. Environmental factors might influence the oxidative conditions in vivo and affect the glycation process as well. Unfortunately, further evaluation of glycation pathways in patients suffering from SLE is needed. It is not known which exact compounds contribute to the increase in concentration of total AGEs in the blood or which ones could be considered potential markers of the glycation process in SLE. Due to the heterogeneous nature of AGEs and the limitations of available analysis techniques, currently, there are no standardized procedures. Thus, the use of AGEs in diagnostics is limited [58]. A study on precursors of AGEs in SLE patients conducted by our team is in progress.

## 5. Conclusions

In conclusion, women suffering from SLE are at risk of intensified glycation and exhibit a deficiency in sRAGE. There were no statistically significant differences between the groups in blood concentrations of CML, CEL, and pentosidine. Smoking might affect the glycation process, and smoking cessation is advised in SLE patients.

## Figures and Tables

**Table 1:** Characteristics of the groups included in the study.

Parameter	SLE Patients (*n* = 31)	Control Group (*n* = 26)	Statistical Significance
mean age (±SD) (years)	56.39 (±11.36)	51.88 (±11.05)	*p* = 0.14
current or past regular smokers % (*n*)	54.84 (17)	26.92 (7)	*p* < 0.05
mean time of smoking (±SD) (years)	23.00 (±11.16)	16.71 (±10.61)	*p* = 0.31
passive smokers % (*n*)	41.94 (13)	26.92 (7)	*p* = 0.24
drinking alcohol % (*n*)	61.29 (19)	84.62 (22)	*p* = 0.05
mean disease duration (±SD) (years)	12.61 (±8.49)	NA	NA
creatinine (μmol/L)	69.44 (±19.76)	NA	NA
mean GFR (±SD) (mL/min/1.73m^2^)	91.17 (±19.86)	NA	NA
mean SLEDAI-2K score (±SD)	11.45 (±7.28)	NA	NA
medication for SLE			
receiving any medication or SLE % (*n*)	90.32 (28)	NA	NA
antimalarial % (*n*) (chloroquine, hydroxychloroquine)	22.58 (7)	NA	NA
corticosteroids % (*n*) (methylprednisolone, prednisone)	35.48 (11)	NA	NA
antimetabolites % (*n*) (azathioprine, methotrexate)	45.16 (14)	NA	NA
manifestations incidence since the time of diagnosis			
rash % (*n*)	93.55 (29)	NA	NA
photosensitivity % (*n*)	80.65 (25)	NA	NA
oral ulcers % (*n*)	25.81 (8)	NA	NA
nonerosive arthritis % (*n*)	90.32 (28)	NA	NA
pleuritis or pericarditis % (*n*)	0 (0)	NA	NA
renal disorder % (*n*)	6.45 (2)	NA	NA
neurologic disorder % (*n*)	3.23 (1)	NA	NA
hematologic disorder % (*n*)	45.16 (14)	NA	NA
cardiovascular disorder % (*n*)	80.65 (25)	NA	NA
immunological disorder /ANA % (*n*)	93.55 (29)	NA	NA

ANA—antinuclear antibodies; GFR—glomerular filtration rate; NA—not applicable or not available; SD—standard deviation; SLEDAI-2K—Systemic Lupus Erythematosus Disease Activity Index 2000.

**Table 2:** Results of the ELISA assay and their statistical significance.

Mean Concentration (±SD)	SLE Patients (*n* = 31)	Control Group (*n* = 26)	Statistical Significance
AGEs (µg/mL)	30.51 (±6.80)	24.02 (±8.50)	*p* < 0.01
CML (µg/mL)	0.31 (±0.09)	0.30 (±0.16)	*p* = 0.65
CEL (µg/mL)	17.73 (±10.66)	19.11 (±7.94)	*p* = 0.35
pentosidine (ng/mL)	4.54 (±1.84)	6.71 (±3.360)	*p* = 0.06
sRAGE (pg/mL)	36.36 (±15.71)	47.18 (±19.41)	*p* < 0.05

SD—standard deviation.

## Data Availability

All obtained data are presented in the article and in the Supplementary materials.

## Supplementary Materials

The following are available online at www.mdpi.com/article/10.3390/cells10123523/s1, Figure S1: Smoking habits in the SLE patients group and the healthy control.

## References

[1] Somers E.C., Marder W., Cagnoli P., Lewis E.E., DeGuire P., Gordon C., Helmick C.G., Wang L., Wing J.J., Dhar J.P. (2014). Population-based incidence and prevalence of systemic lupus erythematosus: The Michigan lupus epidemiology and surveillance program. Arthritis Rheumatol..

[2] Cooper G.S., Bynum M.L.K., Somers E.C. (2009). Recent insights in the epidemiology of autoimmune diseases: Improved prevalence estimates and understanding of clustering of diseases. J. Autoimmun..

[3] Costenbader K.H., Feskanich D., Stampfer M.J., Karlson E.W. (2007). Reproductive and menopausal factors and risk of systemic lupus erythematosus in women. Arthritis Rheum..

[4] Catalina M.D., Owen K.A., Labonte A.C., Grammer A.C., Lipsky P.E. (2020). The pathogenesis of systemic lupus erythematosus: Harnessing big data to understand the molecular basis of lupus. J. Autoimmun..

[5] Martens H.A., Nienhuis H.L.A., Gross S., Van Der Steege G., Brouwer E., Berden J.H.M., De Sévaux R.G.L., Derksen R.H.W.M., Voskuyl A.E., Berger S.P. (2012). Receptor for advanced glycation end products (RAGE) polymorphisms are associated with systemic lupus erythematosus and disease severity in lupus nephritis. Lupus.

[6] Bates M.A., Brandenberger C., Langohr I., Kumagai K., Harkema J.R., Holian A., Pestka J.J. (2015). Silica triggers inflammation and ectopic lymphoid neogenesis in the lungs in parallel with accelerated onset of systemic autoimmunity and glomerulonephritis in the lupus-prone NZBWF1 mouse. PLoS ONE.

[7] Speyer C.B., Costenbader K.H. (2018). Cigarette smoking and the pathogenesis of systemic lupus erythematosus. Expert Rev. Clin. Immunol..

[8] Barbhaiya M., Costenbader K.H. (2016). Environmental exposures and the development of systemic lupus erythematosus. Curr. Opin. Rheumatol..

[9] Tsokos G.C. (2011). Mechanisms of disease: Systemic lupus erythematosus. N. Engl. J. Med..

[10] Yaniv G., Twig G., Shor D.B.A., Furer A., Sherer Y., Mozes O., Komisar O., Slonimsky E., Klang E., Lotan E. (2015). A volcanic explosion of autoantibodies in systemic lupus erythematosus: A diversity of 180 different antibodies found in SLE patients. Autoimmun. Rev..

[11] Nienhuis H.L., de Leeuw K., Bijzet J., Smit A., Schalkwijk C.G., Graaff R., Kallenberg C.G., Bijl M. (2008). Skin autofluorescence is increased in systemic lupus erythematosus but is not reflected by elevated plasma levels of advanced glycation endproducts. Rheumatology.

[12] Aguirre-Valencia D., Suárez-Avellaneda A., Ocampo-Piraquive V., Posso-Osorio I., Naranjo-Escobar J., Nieto-Aristizábal I., Tobón G.J., Cañas C.A. (2019). Mortality in patients with systemic lupus erythematosus in Colombia: A case series. Clin. Rheumatol..

[13] Tselios K., Gladman D.D., Sheane B.J., Su J., Urowitz M. (2019). All-cause, cause-specific and age-specific standardised mortality ratios of patients with systemic lupus erythematosus in Ontario, Canada over 43 years (1971–2013). Ann. Rheum. Dis..

[14] Ene C.D., Georgescu S.R., Tampa M., Matei C., Mitran C.I., Mitran M.I., Penescu M.N., Nicolae I. (2021). Cellular response against oxidative stress, a novel insight into lupus nephritis pathogenesis. J. Pers. Med..

[15] Islam S., Mir A.R., Abidi M., Talha M., Zafar A., Habib S., Moinuddin (2018). Methylglyoxal modified IgG generates autoimmune response in rheumatoid arthritis. Int. J. Biol. Macromol..

[16] Sirois C.M., Jin T., Miller A.L., Bertheloot D., Nakamura H., Horvath G.L., Mian A., Jiang J., Schrum J., Bossaller L. (2013). RAGE is a nucleic acid receptor that promotes inflammatory responses to DNA. J. Exp. Med..

[17] Jyoti, Mir A.R., Habib S., Siddiqui S.S., Ali A., Moinuddin (2016). Neo-epitopes on methylglyoxal modified human serum albumin lead to aggressive autoimmune response in diabetes. Int. J. Biol. Macromol..

[18] Henning C., Glomb M.A. (2016). Pathways of the Maillard reaction under physiological conditions. Glycoconj. J..

[19] Nigro C., Leone A., Fiory F., Prevenzano I., Nicolò A., Mirra P., Beguinot F., Miele C. (2019). Dicarbonyl stress at the crossroads of healthy and unhealthy aging. Cells.

[20] Henning C., Liehr K., Girndt M., Ulrich C., Glomb M.A. (2018). Analysis and chemistry of novel protein oxidation markers in vivo. J. Agric. Food Chem..

[21] Vytášek R., Šedová L., Vilím V. (2010). Increased concentration of two different advanced glycation end products detected by enzyme immunoassays with new monoclonal antibodies in sera of patients with rheumatoid arthritis. BMC Musculoskelet. Disord..

[22] Chen D.Y., Chen Y.M., Lin C.C., Hsieh C.W., Wu Y.C., Hung W.T., Chen H.H., Lan J.L. (2015). The potential role of advanced glycation end products (AGEs) and soluble receptors for AGEs (sRAGE) in the pathogenesis of adult-onset Still’s disease. BMC Musculoskelet. Disord..

[23] De Groot L., Posthumus M.D., Kallenberg C.G.M., Bijl M. (2010). Risk factors and early detection of atherosclerosis in rheumatoid arthritis. Eur. J. Clin. Investig..

[24] Prasad C., Davis K.E., Imrhan V., Juma S., Vijayagopal P. (2019). Advanced glycation end products and risks for chronic diseases: Intervening through lifestyle modification. Am. J. Lifestyle Med..

[25] Gopal P., Reynaert N.L., Scheijen J.L.J.M., Engelen L., Schalkwijk C.G., Franssen F.M.E., Wouters E.F.M., Rutten E.P.A. (2014). Plasma advanced glycation end products and skin autofluorescence are increased in COPD. Eur. Respir. J..

[26] Fishman S.L., Sonmez H., Basman C., Singh V., Poretsky L. (2018). The role of advanced glycation end products in the development of coronary artery disease in patients with and without diabetes mellitus: A review. Mol. Med..

[27] De Leeuw K., Graaff R., de Vries R., Dullaart R.P., Smit A.J., Kallenberg C.G., Bijl M. (2007). Accumulation of advanced glycation endproducts in patients with systemic lupus erythematosus. Rheumatology.

[28] Rodríguez-García J., Requena J.R., Rodríguez-Segade S. (1998). Increased concentrations of serum pentosidine in rheumatoid arthritis. Clin. Chem..

[29] Zieman S.J., Melenovsky V., Clattenburg L., Corretti M.C., Capriotti A., Gerstenblith G., Kass D.A. (2007). Advanced glycation endproduct crosslink breaker (alagebrium) improves endothelial function in patients with isolated systolic hypertension. J. Hypertens..

[30] Dobi A., Bravo S.B., Veeren B., Paradela-Dobarro B., Álvarez E., Meilhac O., Viranaicken W., Baret P., Devin A., Rondeau P. (2019). Advanced glycation end products disrupt human endothelial cells redox homeostasis: New insights into reactive oxygen species production. Free Radic. Res..

[31] Tan K.C.B., Chow W.S., Ai V.H.G., Metz C., Bucala R., Lam K.S.L. (2002). Advanced glycation end products and endothelial dysfunction in type 2 diabetes. Diabetes Care.

[32] Deluyker D., Evens L., Bito V. (2017). Advanced glycation end products (AGEs) and cardiovascular dysfunction: Focus on high molecular weight AGEs. Amino Acids.

[33] Oh S., Son M., Choi J., Lee S., Byun K. (2018). sRAGE prolonged stem cell survival and suppressed RAGE-related inflammatory cell and T lymphocyte accumulations in an Alzheimer’s disease model. Biochem. Biophys. Res. Commun..

[34] Lange J.N., Wood K.D., Knight J., Assimos D.G., Holmes R.P. (2012). Glyoxal formation and its role in endogenous oxalate synthesis. Adv. Urol..

[35] Lee H.T., Wu T.H., Lin C.S., Lee C.S., Wei Y.H., Tsai C.Y., Chang D.M. (2016). The pathogenesis of systemic lupus erythematosus—From the viewpoint of oxidative stress and mitochondrial dysfunction. Mitochondrion.

[36] Shah D., Mahajan N., Sah S., Nath S.K., Paudyal B. (2014). Oxidative stress and its biomarkers in systemic lupus erythematosus. J. Biomed. Sci..

[37] Smallwood M.J., Nissim A., Knight A.R., Whiteman M., Haigh R., Winyard P.G. (2018). Oxidative stress in autoimmune rheumatic diseases. Free Radic. Biol. Med..

[38] Nasiri R., Field M.J., Zahedi M., Moosavi-Movahedi A.A. (2012). Comparative DFT study to determine if α-oxoaldehydes are precursors for pentosidine formation. J. Phys. Chem. A.

[39] Raupbach J., Ott C., Koenig J., Grune T. (2020). Proteasomal degradation of glycated proteins depends on substrate unfolding: Preferred degradation of moderately modified myoglobin. Free Radic. Biol. Med..

[40] Bayoumy N., El-Shabrawi M., Nada H. (2013). A soluble receptor for advanced glycation end product levels in patients with systemic lupus erythematosus. Turkish J. Rheumatol..

[41] Ma C.Y., Ma J.L., Jiao Y.L., Li J.F., Wang L.C., Yang Q.R., You L., Cui B., Chen Z.J., Zhao Y.R. (2012). The plasma level of soluble receptor for advanced glycation end products is decreased in patients with systemic lupus erythematosus. Scand. J. Immunol..

[42] Bobek D., Grčević D., Kovačić N., Lukić K.K., Jelušić M. (2014). The presence of high mobility group box-1 and soluble receptor for advanced glycation end products in juvenile idiopathic arthritis and juvenile systemic lupus erythematosus. Pediatr. Rheumatol..

[43] Tang K.T., Hsieh T.Y., Chao Y.H., Lin M.X., Chen Y.H., Chen D.Y., Lin C.C. (2017). Plasma levels of high-mobility group box 1 and soluble receptor for advanced glycation end products in primary antiphospholipid antibody syndrome patients. PLoS ONE.

[44] Yu S.L., Wong C.K., Szeto C.C., Li E.K., Cai Z., Tam L.S. (2015). Members of the receptor for advanced glycation end products axis as potential therapeutic targets in patients with lupus nephritis. Lupus.

[45] Abou-Raya A.N., Kamel M.A.N., Sayed E.A.G., El-Sharkawy A.A.H. (2016). The plasma level of soluble receptor for advanced glycation end products in systemic lupus erythematosus patients and its relation to disease activity. Alexandria J. Med..

[46] Manganelli V., Truglia S., Capozzi A., Alessandri C., Riitano G., Spinelli F.R., Ceccarelli F., Mancuso S., Garofalo T., Longo A. (2019). Alarmin HMGB1 and soluble RAGE as new tools to evaluate the risk stratification in patients with the antiphospholipid syndrome. Front. Immunol..

[47] Chavakis T., Bierhaus A., Nawroth P.P. (2004). RAGE (receptor for advanced glycation end products): A central player in the inflammatory response. Microbes Infect..

[48] Munguia-Realpozo P., Mendoza-Pinto C., Sierra Benito C., Escarcega R.O., Garcia-Carrasco M., Mendez Martinez S., Etchegaray Morales I., Galvez Romero J.L., Ruiz-Arguelles A., Cervera R. (2019). Systemic lupus erythematosus and hypertension. Autoimmun. Rev..

[49] Zeller C., Appenzeller S. (2008). Cardiovascular disease in systemic lupus erythematosus: The role of traditional and lupus related risk factors. Curr. Cardiol. Rev..

[50] Van Waateringe R.P., Mook-Kanamori M.J., Slagter S.N., Van Der Klauw M.M., Van Vliet-Ostaptchouk J.V., Graaff R., Lutgers H.L., Suhre K., El-Din Selim M.M., Mook-Kanamori D.O. (2017). The association between various smoking behaviors, cotinine biomarkers and skin autofluorescence, a marker for advanced glycation end product accumulation. PLoS ONE.

[51] Mesaros C., Arora J.S., Wholer A., Vachani A., Blair I.A. (2012). 8-oxo-2′-deoxyguanosine as a biomarker of tobacco-smoking-induced oxidative stress. Free Radic. Biol. Med..

[52] Lee H.T., Lin C.S., Lee C.S., Tsai C.Y., Wei Y.H. (2014). Increased 8-hydroxy-2′-deoxyguanosine in plasma and decreased mRNA expression of human 8-oxoguanine DNA glycosylase 1, anti-oxidant enzymes, mitochondrial biogenesis-related proteins and glycolytic enzymes in leucocytes in patients with systemic lupus ery. Clin. Exp. Immunol..

[53] Reynolds P.R., Kasteler S.D., Cosio M.G., Sturrock A., Huecksteadt T., Hoidal J.R. (2008). RAGE: Developmental expression and positive feedback regulation by Egr-1 during cigarette smoke exposure in pulmonary epithelial cells. Am. J. Physiol. Lung Cell. Mol. Physiol..

[54] Wang J., Kay A.B., Fletcher J., Formica M.K., McAlindon T.E. (2009). Alcohol consumption is not protective for systemic lupus erythematosus. Ann. Rheum. Dis..

[55] Wang J., Pan H.F., Ye D.Q., Su H., Li X.P. (2008). Moderate alcohol drinking might be protective for systemic lupus erythematosus: A systematic review and meta-analysis. Clin. Rheumatol..

[56] Barbhaiya M., Lu B., Sparks J.A., Malspeis S., Chang S.C., Karlson E.W., Costenbader K.H. (2017). Influence of alcohol consumption on the risk of systemic lupus erythematosus among women in the Nurses’ Health Study Cohorts. Arthritis Care Res..

[57] Cozier Y.C., Barbhaiya M., Castro-Webb N., Conte C., Tedeschi S.K., Leatherwood C., Costenbader K.H., Rosenberg L. (2019). Relationship of cigarette smoking and alcohol consumption to incidence of systemic lupus erythematosus in a prospective cohort study of black women. Arthritis Care Res..

[58] Perrone A., Giovino A., Benny J., Martinelli F. (2020). Advanced glycation end products (AGEs): Biochemistry, signaling, analytical methods, and epigenetic effects. Oxid. Med. Cell. Longev..

