# Supplementary figures and images for: Advanced Glycation End-Products (AGEs) and Their Soluble Receptor (sRAGE) in Women Suffering from Systemic Lupus Erythematosus (SLE)
Source: Cells. 2021 Dec 13;10(12):3523. doi: 10.3390/cells10123523 (PMC8700453; doi:10.3390/cells10123523)

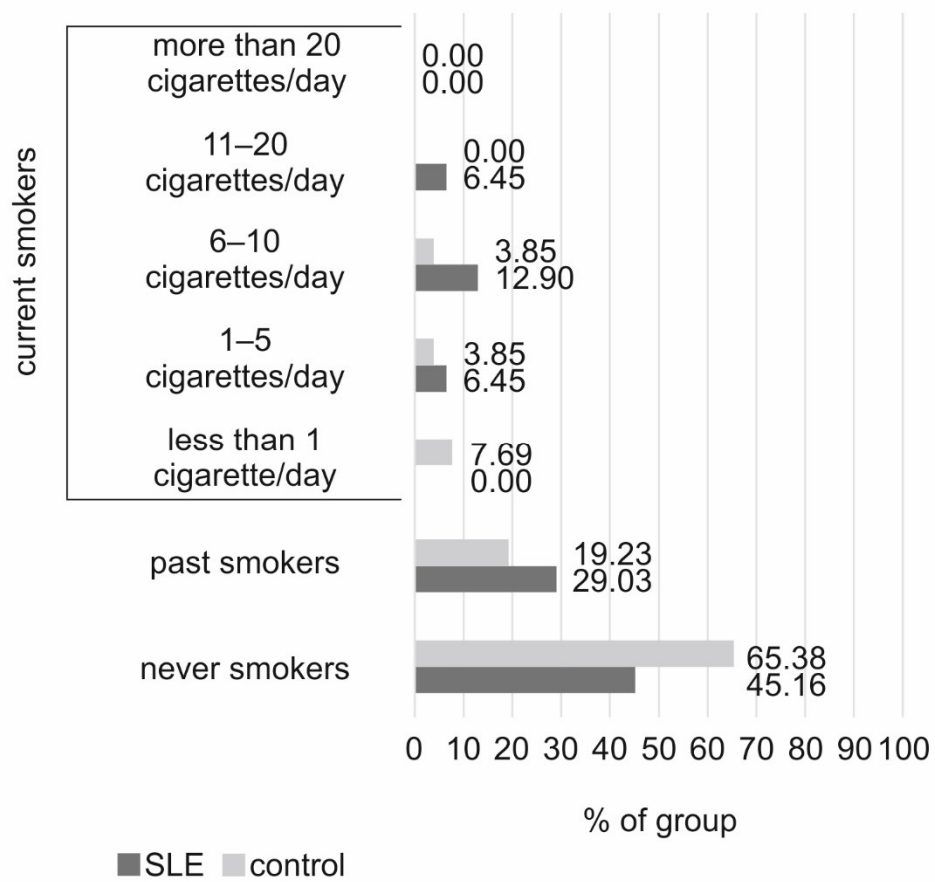

**Figure S1.** Smoking habits in the SLE patients group and the healthy control.

Supplement: Supplementary file 1 [file cells-10-03523-s001.zip › cells-1443593-supplementary.pdf]
